# Cysteine Proteinase C1A Paralog Profiles Correspond with Phylogenetic Lineages of Pathogenic Piroplasmids

**DOI:** 10.3390/vetsci5020041

**Published:** 2018-04-17

**Authors:** Mariano E. Ascencio, Monica Florin-Christensen, Choukri B. Mamoun, William Weir, Brian Shiels, Leonhard Schnittger

**Affiliations:** 1 Instituto de Patobiología, Centro de Investigaciones en Ciencias Veterinarias y Agronómicas (CICVyA), INTA-Castelar, Los Reseros y Nicolas Repetto s/n, Hurlingham 1686, Argentina; ascencio.mariano@inta.gob.ar (M.E.A.); jacobsen.monica@inta.gob.ar (M.F.-C.); 2 National Council of Scientific and Technological Research (CONICET), Ciudad Autónoma de Buenos Aires C1033AAJ, Argentina; 3 Department of Internal Medicine, Section of Infectious Diseases, Yale School of Medicine, New Haven, CT 06520, USA; choukri.benmamoun@yale.edu; 4 Institute of Biodiversity, Animal Health and Comparative Medicine, College of Medical, Veterinary & Life Sciences, University of Glasgow, 464 Bearsden Road, Glasgow G61 1QH, UK; william.weir@glasgow.ac.uk (W.W.); brian.shiels@glasgow.ac.uk (B.S.)

**Keywords:** cysteine proteinases, C1A cysteine-proteinases, piroplasmids, *Theileria*, *Babesia*, *Cytauxzoon*, taxonomy, molecular phylogeny, comparative genomics, evolutionary genomics

## Abstract

Piroplasmid parasites comprising of *Babesia*, *Theileria*, and *Cytauxzoon* are transmitted by ticks to farm and pet animals and have a significant impact on livestock industries and animal health in tropical and subtropical regions worldwide. In addition, diverse *Babesia* spp. infect humans as opportunistic hosts. Molecular phylogeny has demonstrated at least six piroplasmid lineages exemplified by *B. microti*, *B. duncani*, *C. felis*, *T. equi*, *Theileria* sensu stricto (*T. annulata*, *T. parva*, and *T. orientalis*) and *Babesia* sensu stricto (*B. bovis*, *B. bigemina*, and *B. ovis*). C1A cysteine-proteinases (C1A-Cp) are papain-like enzymes implicated in pathogenic and vital steps of the parasite life cycle such as nutrition and host cell egress. An expansion of C1A-Cp of *T. annulata* and *T. parva* with respect to *B. bovis* and *B. ovis* was previously described. In the present work, C1A-Cp paralogs were identified in available genomes of species pertaining to each piroplasmid lineage. Phylogenetic analysis revealed eight C1A-Cp groups. The profile of C1A-Cp paralogs across these groups corroborates and defines the existence of six piroplasmid lineages. *C. felis*, *T. equi* and *Theileria* s.s. each showed characteristic expansions into extensive families of C1A-Cp paralogs in two of the eight groups. Underlying gene duplications have occurred as independent unique evolutionary events that allow distinguishing these three piroplasmid lineages. We hypothesize that C1A-Cp paralog families may be associated with the advent of the schizont stage. Differences in the invertebrate tick host specificity and/or mode of transmission in piroplasmid lineages might also be associated with the observed C1A-Cp paralog profiles.

## 1. Introduction

Piroplasmids are tick-transmitted obligatory hemoprotozoan parasites including species of the genus *Babesia*, *Theileria*, and *Cytauxzoon.* Many species are of veterinary significance, either due to their negative economic impact on livestock industries or due to the diseases they cause in pets and occasionally in wild life [1,2,3]. Examples of the former group include *Babesia bovis* and/or *B. bigemina* causing bovine babesiosis of cattle; *Theileria annulata*, *Theileria parva*, and *Theileria orientalis* that cause tropical theileriosis, East Coast Fever, and oriental theileriosis of cattle, respectively; and *Babesia ovis* causing ovine babesiosis of sheep [4,5,6,7,8]. Piroplasmids that infect companion animals include *Cytauxzoon felis*, the causative agent of feline cytauxzoonosis, and *Theileria equi* and *Babesia caballi* causing equine piroplasmosis [9,10,11,12]. In addition, a third group of piroplasmid species has been reported as pathogens of human babesiosis including *B. microti*, *B. duncani*, and *B. venatorum*, while *B. divergens* infects both cattle and humans [2,13,14,15,16].

Classical taxonomy distinguishes four piroplasmid genera based on morphology and/or biological characteristics of their life cycle history. (i) *Babesia* sensu stricto (s.s.) shows typically a transovarial transmission by *Rhipicephalus* or *Hyalomma* ticks; (ii) *Theileria* sensu stricto (s.s.) and (iii) *Cytauxzoon* spp. are typically transstadially transmitted, the former by *Haemaphysalis* and *Hyalomma* ticks and the latter by *Dermacentor* and *Amblyomma* ticks. In addition, *Theileria* and *Cytauxzoon* are characterized by the presence of an additional intraleukocytic schizont stage, which in *Theileria* is present in macrophages and T and B lymphocytes whereas in *Cytauxzoon* in mononuclear phagocytes. Finally, a fourth piroplasmid group is referred to as (iv) *Babesia* sensu lato (s.l.) which are commonly transmitted transstadially by ticks of the genus *Ixodes* but cannot be classified as *Theileria* (s.s.) since schizonts are absent [17,18,19].

Importantly, molecular phylogenetic analysis based on the 18S rRNA gene has been able to largely confirm the above outlined classical taxonomic units, yet could further refine and improve their relationship resulting in the definition of six piroplasmid genera [2,20]. Nearly complete agreement between classical taxonomy and molecular phylogeny has been shown for the *Babesia* s.s. and *Theileria* s.s. groups (Clades VI and V, respectively, as defined by Schnittger et al. [2]). It is however noteworthy that it could be shown that *Theileria equi* does not belong to *Theileria* s.s. but represents with *B. bicornis* a separate monophyletic group (Clade IV as defined by Schnittger et al. [2]), a finding that was subsequently confirmed by whole genome and mitochondrial gene analysis [21,22]. Furthermore, findings based on 18S rRNA phylogenetic analysis strongly suggests that the genus *Cytauxzoon* represents a monophyletic group that can be clearly delineated from *Theileria* s.s. (Clade IIIb as defined by Schnittger et al. [2]). However, using mitochondrial genes, Schreeg et al. [22] could integrate *C. felis* as sister group of *Theileria* s.s. into a single taxon with good support. By molecular phylogeny it was possible to show that *Babesia* s.l. parasites represent a paraphyletic group which is distantly related to *Babesia* s.s. and more closely related to *Theileria* and *Cytauxzoon* parasites. Furthermore, the *Babesia* s.l. group can be distinguished into at least two genera (Clade I and Clade II as defined by Schnittger et al. [2], Lack et al. [20], Schreeg et al. [22]). Meanwhile, it is worth noting that additional piroplasmid genera have been identified that are not considered in the present study as no genome sequences are available from species pertaining to these groups [23,24].

Cysteine proteinases (Cps) are involved in vital steps of the apicomplexan life cycle such as host cell invasion, egress, and parasite nutrition [25,26,27]. Papain-like cysteine peptidases designated as C1A family (C1 family of the Clan CA) attracted particular interest because of their involvement in pathogenic processes such as intracellular hemoglobin degradation and cytoskeletal rupture during host cell egress, as described for falcipain of *Plasmodium falciparum* [28,29,30].

C1A falcipain-homologs bovipain, ovipain, and babesipain of *B. bovis*, *B. ovis*, and *B. bigemina*, respectively, have been characterized [31,32,33,34]. Interestingly, whereas only a single bovipain and ovipain encoding gene is present in the *B. bovis* and *B. ovis*-genome, this gene has expanded in a gene family encoding six and seven paralogs in *T. parva* and *T. annulata*, respectively [31,35,36]. It has been suggested that the augmented copy number of falcipain-orthologs in *T. annulata* and *T. parva* reflects the biological differences of this piroplasmid lineage compared to *B. bovis* such as the distinct tick host specificity, transstadial vs. transovarial transmission, and presence vs. absence of the schizont parasite stage [36,37,38].

In this study, all C1A cysteine proteinases (C1A-Cp) were mined from available genomes of pathogenic *Babesia*, *Theileria*, and *Cytauxzoon* piroplasmids appertaining to Clades I to VI as defined by Schnittger et al. [2]. A phylogenetic tree was constructed to allocate C1A-Cp paralogs into different evolutionary groups (C1A-Cp Groups 1–8). We hypothesized that the profile of C1A-Cp paralogs across revealed evolutionary group is specific and corresponds with the phylogenetic classification of piroplasmids into Clades I to VI, as previously suggested.

## 2. Materials and Methods

The amino acid sequence of C1A family holotype, Peptidase_C1 (PF00112, Pfam), was used in a BLASTp search adjusting parameter settings to piroplasmid sequences (taxid:5863) and *refseq* database in order to identify homologs in completely sequenced genomes of piroplasmid species from *B. bigemina* strain BOND [39], *B. bovis* strain T2Bo [40], *B. microti* strain RI [41], *T. annulata* strain Ankara [42], *T. equi* strain WA [21], *T. orientalis* strain Shintoku [43], and *T. parva* strain Muguga, [44]. Since the *C. felis* genome is not available in the GenBank, corresponding C1A-Cp have been retrieved using a similar approach from the PiroplasmaDB database (*C. felis* strain Winnie [45]). In addition, C1A-Cp sequences of *B. duncani* and *B. ovis* have been retrieved by courtesy from as yet public unavailable genomes (*B. duncani* strain WA1: Choukri Ben Mamoun, Yale School of Medicine, New Haven, CT, USA; *B. ovis* strain Israel: William Weir, University of Glasgow, Glasgow, UK). Following the above described procedure, all sequence hits with an *E*-value < 0.05 were subsequently included in the study. The C1A-Cp proteinase domain of each sequence was determined either by MEROPS (http://merops.sanger.ac.uk) and/or Pfam (http://pfam.xfam.org) and then aligned using the MUSCLE algorithm. To eliminate end gaps, the alignment was finally truncated at its C and N terminal to a length of 315 aa. The phylogenetic tree was constructed using maximum likelihood based on the model LG [46]. Briefly, initial tree(s) for the heuristic search were obtained by applying the Neighbor-Joining method to a matrix of pairwise distances estimated using a JTT model. Then, the best evolutionary model, determined based on Akaike Information Criteria (AIC) as LG + G (G = 1.4880, estimated over five categories) was applied to infer the tree. The analysis involved eighty-three amino acid sequences, of which eighty-two belong to C1A-Cp of *Babesia*, *Theileria*, and *Cytauxzoon* spp., and falcipain-2 of *P. falciparum* was used as an outgroup. Altogether 1000 bootstrap replicates were carried out. Bootstrap values ≥80 were considered as highly significant. Alignment, model search, and tree construction was carried out using MEGA 6 [47]. Orthology was inferred by bidirectional best hits (BBH) in pairwise genome comparisons using pBLAST. However, as the *C. felis*, *B. ovis* and *B. duncani* genomes are not available in GenBank, BBH could not be carried out for these species.

## 3. Results and Discussion

### 3.1. Molecular Phylogeny Allows Subgrouping of Piroplasmid C1A Cysteine Proteinases

The aim of this work was to compare C1A-Cp homologs across genomes of well-defined and evolutionary diverse piroplasmid species to assess whether paralog numbers and variant profiles support current piroplasmid taxonomy.

**Figure 1:**
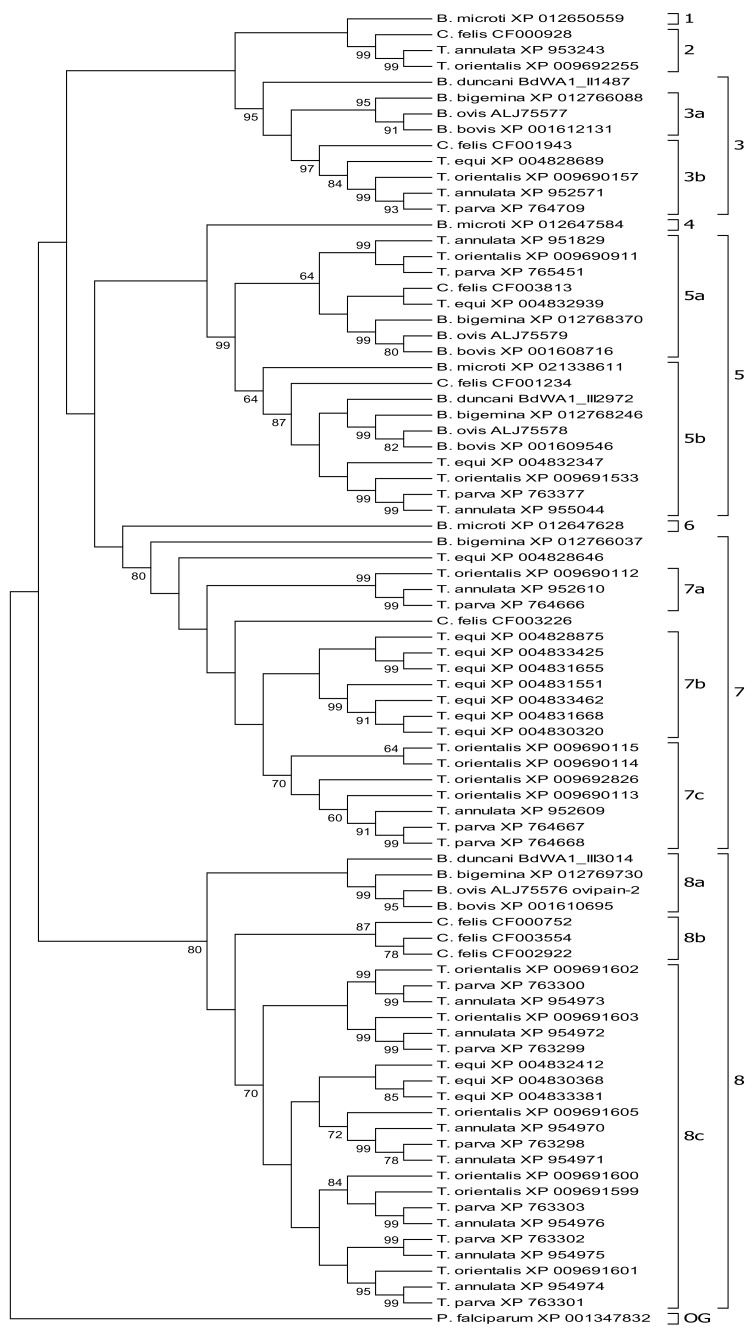
Maximum likelihood tree of C1A proteinase amino acid domains of *Babesia*, *Theileria*, and *Cytauxzoon* parasites. Each C1A cysteine proteinase sequence is designated with the corresponding piroplasmid species and accession number. Numbers 1–8 designate revealed C1A cysteine proteinase Groups 1–8. OG, outgroup. Bootstrap values based on 1000 replicates are displayed next to the branches.

A total of eighty-two cysteine proteinase-homologs (including orthologs and paralogs) of the subfamily C1A were identified in the ten studied piroplasmid genomes appertaining to six different evolutionary lineages (Table 1). Sixty-six of the eighty-two C1A-Cp displayed the conserved functional amino acids glutamine, cysteine, histidine, and asparagine at catalytic active sites, whereas fourteen showed substitutions and/or gaps at these positions representing enzymatically non-functional proteinase homologs. Based on the inferred phylogenetic tree, C1A-Cp could be divided in altogether eight groups designated, Groups 1–8 (Figure 1). In addition to the displayed maximum likelihood tree, a neighbor joining tree was also inferred resulting in similar groupings. Noteworthy these groupings are neither recognized by MEROPS nor by Pfam evidencing the sensitivity of our approach.

### 3.2. Molecular Phylogeny Provides Evidence for Independent Duplication Events of C1A-Cp in Different Piroplasmid Lineages

In the phylogenetic tree, at least four monophyletic lineages of CpA-Cp homologs are strongly supported that precede piroplasmid speciation (Figure 1). Group 1 consists of a single C1A-Cp of *B. microti* which segregates as sister to Group 2, although with a non-significant support. An analogous situation is observed for each *B. microti* C1A-Cp of Groups 4 and 6 which place as sisters in relation to Groups 5 and 7, respectively, likewise with a non-significant bootstrap. We consider each of the *B. microti*-C1A-Cp of Groups 1, 4, and 6 a group of their own, since they neither show a significant relation to each other nor to any other of the remaining groups. We consider that this observation reflects a loss of phylogenetic signal in these sequences supporting the ancient evolutionary relationship of *B. microti* (Clade I) to all other piroplasmid species of Clade II to VI, as has been reported previously [2,20,49]. As *B. microti* sequences were not included in the phylogenetic analysis of Martins et al. [36], corresponding groups do not appear in their study.

Interestingly, in Group 2, the C1A-Cp proteinase of *C. felis* (Clade IIIb) places with strong support as sister to *T. annulata* and *T. orientalis* (*Theileria* s.s., Clade V) sequences. A truncated C1A-Cp of *T. parva* has not been included in the tree analysis, while C1A-Cp of other piroplasmid species might have gone lost in this group. This group is related with the SERA Cp-family from *P. falciparum* of which some members have serine instead cysteine in the active site [50].

Except for *B. microti*, Group 3 includes C1A-Cp of all studied piroplasmid species and is very well supported. C1A-Cps of this group represent orthologs as confirmed by BBH and should be thus suitable to verify the phylogenetic relationship between these species. Importantly, C1A-Cps of *B. duncani* (Clade II), *C. felis* (Clade IIIb), *T. equi* (Clade IV), and *Theileria* s.s. parasites (Clade V) endorse the phylogenetic relation as established by Schnittger et al. [2] with strong support. However, the placement of *Babesia* s.s. proteinases (Group 3a, Clade VI) as sister group with respect to *C. felis* (Clade IIIb), *T. equi* (Clade IV), and *Theileria* s.s. parasites (Clade V) (Group 3b) seems to be incidental and is not supported. Interestingly, C1A-Cp of *T. annulata* (XP952571) and *T. parva* (XP764709) show serine instead of cysteine in their active site as has been also reported for some SERA proteins of *P. falciparum* [36,50]. The proteinases of this subgroup belong to the cathepsin L-like Cps.

Group 5 C1A-Cp are of the cathepsin C type and segregate with a strong bootstrap support. Subgroup 5a includes C1A-Cp of all studied species but *B. microti*. As expected, cysteine proteinases of *Theileria* s.s. parasites (Clade V) place as sister taxon with regard to *Babesia* s.s. (Clade VI) group, whereas the placement of *C. felis* (Clade IIIb) and *T. equi* (Clade IV) is unsupported.

Exclusively, Subgroup 5b includes C1A-Cp orthologs of all ten studied piroplasmids. The tree placement of C1A-Cp of *B. microti* (Clade I), *C. felis* (Clade IIIb), *T. equi* (Clade IV), and *Theileria* s.s. parasites (Clade V) agrees with the phylogenetic relation as proposed by Schnittger et al. [2]. However, the position of *T. equi* (Clade IV) and *B. duncani* (Clade II) are not supported. Thus, not surprisingly, the segregation of *B. duncani* as sister to *Babesia* s.s. (Clade VI) disagrees with the commonly accepted placement of this species. C1A-Cp proteinases of Subgroups 5a and 5b represent out-paralogs with regard to each other as duplication has preceded speciation (Table 2).

C1A-Cp of Group 7 include families of eight, two, three, and five paralogs in *T. equi* (Clade IV), *T. annulata*, *T. parva*, and *T. orientalis* (*Theileria* s.s., Clade V) genomes, respectively, whereas C1A-Cp members of *B. bovis*, *B. ovis*, and *B. duncani* have been lost. Possibly due to multiple events of gene duplication and loss in this sequence group, current evolutionary lineages of some single sequences such as *B. bigemina* (XP_012766037), *T. equi* (XP004828646), and *C. felis* (CF003226) are difficult to interpret based on their tree segregation and low support (Group 7). C1A-Cp within Subgroup 7a and *T. orientalis* XP_009690114, *T. annulata* XP_952609, and *T. parva* XP_764667 within Subgroup 7c represent co-orthologs. Remarkably, after speciation into *T. equi* multiple duplication events have resulted in an extensive in-paralog group of altogether seven C1A-Cp (Subgroup 7b). This gene amplification seems to have occurred as a single unique event in this piroplasmid species which strongly supports its classification separate from *Theileria* s.s. Likewise, multiple duplication events in *T. orientalis* resulted in a group of at least four paralogs (Subgroup 7c). Most likely, *T. orientalis* proteinases XP_009692826 and the lineage resulting in XP_009690114/XP_009690115 have arisen prior to speciation and resulted therefore in the generation of three out-paralogs in *T. orientalis* with respect to orthologs XP_952609 of *T. annulata*, XP_764667 of *T. parva*, and XP_009690114 of *T. orientalis*. In contrast, C1A-Cp pairs of *T. parva* (XP_764667/XP_764668) and of *T. orientalis* (XP_009690114/XP_009690115) represent in-paralogs that have generated after speciation. In addition, C1A-Cps of Group 7, similar to those of Groups 3 and 8 discussed in the following, are of the cathepsin L-like type.

Group 8 is well supported and consists of three monophyletic C1A-Cp lineages. Contrary to the expected, the well supported *Babesia* s.s. (Clade VI) sequences (Subgroup 8a), which include ovipain-2 of *B. ovis* and bovipain-2 of *B. bovis*, place as a sister with respect to the remaining proteinases of Group 8 (Subgroup 8a). Notably, however, the position of the C1A-Cp of *B. duncani* as a sister of this grouping is not supported. Three paralogs of *C. felis* (Subgroup 8b) represent a sister group to *T. equi* (Clade IV) and *Theileria* s.s. (Clade V) sequences (Subgroup 8c). Most likely, C1A proteinases of *C. felis* (Clade IIIb) have expanded to a family of three in-paralogs after the split of this lineage from *T. equi* (Clade IV) and *Theileria* s.s. (Clade V). C1A cysteine proteinases of *T. equi* (Clade IV) but in particular those of *Theileria* s.s. (Clade V) species *T. annulata*, *T. parva*, and *T. orientalis* have greatly expanded into three, seven, six, and six C1A paralog families, respectively (Subgroup 8c). The C1A proteinase family of *T. equi* represent in-paralogs that have generated after the split of this species from *Theileria* s.s. (Subgroup 8c). Based on the observed segregation pattern of *T. annulata*, *T. parva*, and *T. orientalis* C1A-Cp, the underlying gene duplication events most likely happened before speciation in their most recent common ancestor (MRCA). This strongly suggests that the respective C1A-Cp-encoding gene family has been inherited by the descendants of all *Theileria* s.s. parasites and presents a common feature of this group. Thus, in accordance with genome homology nomenclature, each member of a protein family of a given species (e.g., *T. annulata*) represents an out-paralog to any of the other protein family members of the remaining two species (e.g., *T. parva* and *T. orientalis*). The above interpretation is also supported by synteny studies of the respective *T. annulata*, *T. parva*, and *T. orientalis* genome regions [31,35,37,38]. The unique evolution of the C1A-Cp family of *Theileria* s.s. seems to be a characteristic hallmark of this parasite genus that might have been crucial for their evolutionary radiation [51]. In summary, independent and characteristic C1A proteinase duplication events distinguish and define the *T. equi* and the *C. felis* groups from each other but also from *Theileria* s.s., supporting their classification into three evolutionary lineages and taxonomic entities. Furthermore, altogether eight subgroups of C1A-Cp proteinase paralogs could be distinguished, of which the five Groups 2, 3, 5, 7, and 8, were highly significantly supported. Group 2 comprises cysteine proteinases of the SERA-like family, whereas Groups 3, 7, and 8 and Group 5 comprise cysteine proteinases of the cathepsin L and cathepsin C-like family, respectively. In addition, Groups 1, 4, and 6 correspond to three C1A-Cp proteinases of *B. microti* whose assignment to other groups could not be supported reflecting the relatively distant evolutionary relation of *B. microti* with respect to the other piroplasmid lineages included in the study.

Recently, a molecular phylogenetic analysis using concatenated mitochondrial genes *cytb*, *cox1*, and *cox3* and the 18S rRNA gene could integrate *C. felis* with *Theileria* s.s. into a single monophyletic group. In addition, synteny of mitochondrial genomes of *C. felis* corresponded with those of *Theileria* s.s. and *Babesia* s.s. Furthermore, in the inferred tree, *T. equi* represents a sister group with respect to a clade comprised of *C. felis* and *Theileria* s.s. and a clade of *Babesia* s.s. [22]. This is in contrast to the present phylogenetic analysis in which *C. felis*-C1A-Cp segregate commonly with a high bootstrap support as a sister taxon with regard to sequences of *T. equi* and *Theileria* s.s. (see Group 2 and Subgroups 3b, 5b, 7b, and 8b), and, whenever other placements are observed, they remain unsupported (Groups 5a). Furthermore, *T. equi* commonly places as sister with regard to *Theileria* s.s. (Subgroups 3b, 5a, 5b, 7b/c, and 8c), although this position is only supported in a single case (Subgroup 3b). All in all, our findings strongly support *C. felis* as a sister group of *T. equi* and *Theileria* s.s., rather than *T. equi* as a sister group of *C. felis* and *Theileria* s.s., corresponding with previous studies [2]. This analysis also suggests that cysteine proteinase sequences may aid in the phylogenetic inference particularly of more closely related piroplasmid species and/or groups.

### 3.3. C1A Cysteine Proteinase Profiles Characterize Piroplasmid Lineages

A synopsis of the allocation of identified C1A cysteine-proteinases to C1A groups and piroplasmids is shown in Table 2. By far, the highest number of C1A-Cp paralogs was found in *T. equi* (*n* = 13), *T. annulata* (*n* = 13), *T. parva* (*n* = 13), and *T. orientalis* (*n* = 15), followed by *C. felis*, which also presents a relatively high number of C1A-Cp paralogs (*n* = 8). In contrast, the analyzed *Babesia* parasites account for a lower number of 3–5 paralogs. Characteristic C1A-Cp group profiles allow distinguishing between the different piroplasmid Clades I to VI. In the case of *B. microti* (Clade I), three of four C1A-Cp are unique and have no orthologs in any other piroplasmid species. *B. duncani* (Clade II) exhibits only a single C1A-Cp in Groups 3, 5, and 8. *C. felis* (Clade IIIb) displays, together with *T. equi* (Clade IV) and *Theileria* s.s. (Clade V), C1A-Cps in Groups 3, 5, 7, and 8, while a C1A-Cp in Group 2 is exclusively found in *C. felis* and *Theileria* s.s. In Group 8 of *C. felis* (Clade IIIb) and *T. equi* (Clade IV), a moderate expansion into three paralogs is observed. In addition, *T. equi* (Clade IV) presents a conspicuously increased number of eight paralogs in Group 7. All studied *Theileria* s.s exhibit characteristic C1A expansions of 2–5 paralogs in Group 7, and of 6–7 paralogs in Group 8. Finally, exclusively in *Babesia* s.s. a single C1A-Cp is found in Group 3 and 8, and a C1A paralog pair in Group 5. The observation that similar C1A paralog profiles repeat in *T. annulata*, *T. parva*, and *T. orientalis* of *Theileria* s.s. (Clade V) and in *B. ovis*, *B. bovis*, and *B. bigemina* of *Babesia* s.s. (Clade VI) suggests that they constitute suitable taxonomic genome markers for the respective groups.

It is noticeable that *C. felis*, *T. equi*, and *Theileria* s.s., which are all characterized by the possession of a schizont stage, jointly exhibit a moderate (*C. felis*) to extreme (*T. equi* and *Theileria* s.s.) expansion of C1A-Cp paralogs. We cautiously suggest that this might be functionally associated with the evolution of the schizont stage. Alternatively, different tick host specificity and/or mode of transmission might also be associated with the observed C1A-Cp paralog profiles. Future investigations including gene knockout studies might be able to shed light on the hypothesized functional associations.

## Figures and Tables

**Table 1:** *Babesia*, *Theileria*, and *Cytauxzoon* parasites included in this study and their classical taxonomy, molecular phylogeny, common designation, diseases, and geographic distribution.

Species	Classic Taxonomy	^3^ Phylogenetic Clade	Common Designation	Diseases	Geographic Distribution
*B. microti*	*Babesia* s.l.	I	*B. microti*-group	human babesiosis	USA, Europe, Japan,
*B. duncani*		II	^4^ western clade	human babesiosis	USA
^1^ *T. bicornis*		IIIa	*Theileria* s.l.	n.d.	Africa
*C. felis*	Cytauxzoon	IIIb	Cytauxzoon	feline cytauxzoonosis	USA
*T. equi*	^2^*Theileria* s.s.	IV	^5^ *T. equi*	^6^ equine piroplasmosis	tropical and subtropical regions worldwide
*T. annulata* *T. parva* *T. orientalis*	*Theileria* s.s.	V	true *Theileria*	tropical theileriosis East Coast Fever oriental theileriosis	tropical and subtropical regions of the old world East Africa Asia
*B. bovis* *B. bigemina* *B. ovis*	*Babesia* s.s.	VI	true *Babesia*	^7^ bovine babesiosis ovine babesiosis	tropical and subtropical regions worldwide

^1^ No sequenced genome is available for any species of Clade IIIa but for the sake of completeness *T. bicornis* has been included in this table; ^2^ *T. equi* has been described by classical taxonomy as *Theileria* s.s. [9]; ^3^ Clades are designated as defined by Schnittger et al. [2]; ^4^ Piroplasmid species that place in this clade have now also been described in other geographic regions besides the US western states Washington and California [48]; ^5^ *T. equi* segregates with *B. bicornis* in a single Clade IV; ^6^ The disease referred to as equine piroplasmosis is caused by infection with *T. equi* and/or *B. caballi*; ^7^ bovine babesiosis is caused by infection with *B. bovis* and/or *B. bigemina*. n.d., no data available.

**Table 2:** Assignment of C1A cysteine proteinase sequences to *Babesia*, *Theileria*, and *Cytauxzoon* species and clades.

^2^ C1A-Cp Group	^1^ Piroplasmid Clade									
	I	II	IIIb	IV	V			VI		
	*B. microti*	*B. duncani*	*C. felis*	*T. equi*	*T. annulata*	*T. parva*	*T. orientalis*	*B. ovis*	*B. bovis*	*B. bigemina*
1	XP_012650559									
2			CF000928		XP_953243	^3^ XP_764233	XP_009692255			
3		BdWA1_II1487	*CF001943*	*XP_004828689*	*XP_952571*	*XP_764709*	*XP_009690157*	ALJ75577	**XP_001612131**	XP_012766088
4	*XP_012647584*									
5	XP_021338611	BdWA1_III2972	CF001234	XP_004832347	XP_955044	XP_763377	XP_009691533	ALJ75578	**XP_001609546**	*XP_012768370*
			CF003813	XP_004832939	XP_951829	XP_765451	XP_009690911	ALJ75579	***XP_001608716***	XP_012768246
6	XP_012647628									
7			CF003226	XP_004828646	**XP_952610**	XP_764666	XP_009690112			
				XP_004828875	**XP_952609**	XP_764667	XP_009690113			XP_012766037
				*XP_004830320*		XP_764668	**XP_009690114**			
				*XP_004831551*			XP_009690115			
				XP_004831655			XP_009692826			
				*XP_004831668*						
				XP_004833425						
				*XP_004833462*						
8		BdWA1_III3014	CF002922	XP_004830368	XP_954970	XP_763298	XP_009691605	ALJ75576	**XP_001610695**	XP_012769730
			CF003554	XP_004832412	**XP_954972**	XP_763299	XP_009691603			
			CF000752	XP_004833381	**XP_954973**	XP_763300	XP_009691602			
					**XP_954974**	XP_763301	XP_009691601			
					**XP_954976**	XP_763303	XP_009691599			
					**XP_954975**	XP 763302	XP_009691600			
					XP_954971					
**∑ 83**	4	3	8	13	13	13	15	4	4	5

^1^ Piroplasmid lineages are designated as defined by Schnittger et al. [2] by roman numbers. ^2^ C1A proteinase groups are designated according to those identified in the phylogenetic tree of Figure 1. ^3^ This C1A proteinase has not been included in the phylogenetic tree since it is truncated but could be confirmed by BBH. C1A proteinases framed blue and green represent in- and out-paralogs, respectively. C1A proteinases designated by an underlined accession numbers within a line represent orthologs as identified either by BBH or by tree analysis with respect to C1A proteinases designated with a bold accession number. C1A proteinases in italics represent non-functional proteinase homologs that exhibit incomplete or exchanged amino acids at active sites.
